# Suicide following acute admissions for physical illnesses across England and Wales

**DOI:** 10.1017/S0033291717001787

**Published:** 2017-07-17

**Authors:** S. E. Roberts, A. John, U. Kandalama, J. G. Williams, R. A. Lyons, K. Lloyd

**Affiliations:** 1 Swansea University Medical School, Singleton Park, Swansea University, Swansea, UK; 2 Farr Institute of Health Informatics Research, Swansea University, Swansea, UK

**Keywords:** Acute admissions, physical illnesses, suicide

## Abstract

**Background:**

The study aim was to establish and quantify suicide risk following acute admissions for all major physical illnesses, for confirmatory purposes, from two independent information sources from different countries.

**Methods:**

Record linkage of inpatient and death certificate data for 11 004 389 acute admissions for physical illnesses in England and 713 496 in Wales. The main outcome measure was standardised mortality ratios (SMRs) for suicide at 1 year following discharge from hospital.

**Results:**

There were 1781 suicides within 1 year of discharge in England (SMR = 1.7; 95% = 1.6–1.8) and 131 in Wales (SMR = 2.0; 1.7–2.3). Of 48 major physical illnesses that were associated with at least eight suicides in either country, there was high consistent suicide mortality (significant SMR >3) in both countries for constipation (SMR = 4.1 in England, 7.5 in Wales), gastritis (4.4 and 4.9) and upper gastrointestinal bleeding (3.4 and 4.5). There was high suicide mortality in one country for alcoholic liver disease, other liver disease and chronic pancreatitis; for epilepsy and Parkinson's disease; for diabetes, hypoglycaemia and hypo-osmolality & hyponatraemia; and for pneumonia, back pain and urinary tract infections.

**Conclusions:**

There is little or no increased suicide mortality following acute admissions for most physical illnesses. Much of the increased suicide mortality relates to gastrointestinal disorders that are often alcohol related or specific chronic conditions, which may be linked to side effects from certain therapeutic medications. Acute hospital admissions for physical illnesses may therefore provide an opportunity for targeted suicide prevention among people with certain conditions, particularly alcohol related disorders.

## Introduction

Globally, suicide is a major public health issue with 800 000 deaths every year (World Health Organisation, 2017). Premature loss of life in early and mid-adulthood also makes suicide an important global cause of years of healthy life lost (Lozano *et al*. 2013). In the UK, there are approximately 6000 suicides annually, of which approximately 25% occur within 1 year of an acute hospital admission (Office for National Statistics, 2014; study data).

Established risk factors for suicide include mental disorders such as depression, anxiety and bipolar disorder, as well as alcohol and substance misuse disorders (Newman & Bland, 1991; Angst *et al.* 2005; Merrall *et al.* 2012). Previous studies of physical illnesses and suicide have been confined mostly to single disorders rather than looking more broadly at the overall relationship. Increased suicide rates have been established for conditions including epilepsy (Hawton *et al.* 1980; Bell *et al.* 2009*a*, *b*; Singhal *et al.* 2014; Erlangsen *et al.* 2015), type I diabetes (Kyvik *et al.* 1994; Singhal *et al.* 2014; Erlangsen *et al.* 2015), multiple sclerosis (Harris & Barraclough, 1994; Manouchehrinia *et al.* 2016), cancers (Harris & Barraclough, 1994; Druss & Pincus, 2000; Crump *et al.* 2014; Erlangsen *et al.* 2015), and asthma (Crump *et al.* 2014; Barker *et al.* 2015), although little has been reported for many other physical illnesses.

Studies that have reported on associations between suicide and a wide range of physical illnesses include cohort studies, based mostly in the community (Webb *et al.* 2012; Crump *et al.* 2014; Erlangsen *et al.* 2015), rather than in hospital settings (Qin *et al.* 2013), and largely from Scandinavia (Qin *et al.* 2013; Crump *et al.* 2014; Erlangsen *et al.* 2015); or based on review methodology (Harris & Barraclough, 1994) with varying methodology used across the reports included. A recent study found that those who died by suicide were more frequently discharged from general rather than psychiatric hospitals (Dougall *et al.* 2014). While this study highlighted that engaging in suicide prevention within the general hospital setting deserves more attention, other than for self harm, it provided little evidence for where these efforts should be focussed. Information on increased risks of suicide among people hospitalised acutely for physical illnesses has not been reported previously and could be useful for suicide prevention purposes and for raising clinical awareness, especially as longer duration contacts with health services provide opportunities for interventions to reduce suicide risks.

The aim of the study was to establish and quantify any increased or decreased risks of suicide following acute inpatient admissions for all major physical illnesses. Importantly, to provide confirmatory evidence, the study was based on two corresponding but independent hospital information sources from two neighbouring countries. The main study hypothesis was that most physical illnesses with previously established links with suicide would also be associated in this study, along with some alcohol-related disorders that have not been reported previously.

## Method

### Study population

A retrospective cohort design was used for people in England (population 53.5 million in 2012) and Wales (3.0 million) who were hospitalised as emergencies. The study was based on national administrative inpatient data; Hospital Episode Statistics in England and the corresponding Patient Episode Database for Wales. The inpatient data were systematically linked to mortality data from the Office for National Statistics and the Welsh Demographic Service to identify all suicides that occurred up to 1 year following discharge from hospital. The data were compiled and accessed through the Secure Anonymised Information Linkage (SAIL) databank (Lyons *et al.* 2009), by a team from the Farr Institute of Health Informatics Research. The ascertainment of mortality has been validated as >98% accurate and the record linkage methodology, based on a unique anonymised, encrypted linking field for each patient, as >99.8% accurate (Lyons *et al.* 2009).

The study population included adults aged 18 years or over, who had an unscheduled admission to any public hospital in England or Wales from 1 January 2004 to 31 December 2011. We included each person's first emergency admission during the study period and followed them up in order to establish all suicides within 1 year of discharge from hospital. Subsequent admissions during the study period were similarly included for 1 year follow-up provided they occurred at least 1 year after hospital discharge from the preceding admission and that the discharge date occurred at least 1 year before the end of the study period. Our study cohorts should not be affected substantially by attrition over the 1-year follow-up period, as annual population emigration from both England and Wales was less than 2.8% during the study period (Office for National Statistics, 2017; Welsh Assembly Government, 2013).

### Physical illnesses

As the focus of the study was on physical rather than mental illnesses, we studied all physical illnesses (ICD-10 codes A00-E99, G01-Q99), based on the principal diagnosis at discharge, excluding mental and behavioural disorders. We included all ‘major’ physical illnesses that led to at least eight suicides within 1 year of discharge from hospital in either England or Wales. In total 48 physical illnesses fulfilled the inclusion and exclusion criteria. ICD-10 codes for each disease are listed in online Supplementary Appendix 1.

### Study outcome measures

The main study outcome measure was the standardised mortality ratio (SMR) to assess suicide mortality up to 1 year following hospital discharge in the study cohorts compared with that in the corresponding general population. Suicide was defined by intentional self harm (ICD-10 codes X60-X84) or undetermined intent (Y10-Y33.8) when recorded as the underlying cause of death on death certificates. Deaths coded as Y33.9 (pending verdicts) were excluded, as a large proportion of these deaths are subsequently determined to be homicides (Griffiths *et al.* 2005).

### Methods of analysis

Age and sex adjusted SMRs for suicide were calculated using the indirect method and by applying age and sex specific suicide mortality in the general adult population of England of Wales to obtain the expected suicides in the study populations and by then comparing the observed and expected suicides. The age groups used were 18–19 years, 20–24 quinquennially up to 80–84 and then 85+ years. For each of the 48 physical illnesses, age and sex were missing in <0.01% of cases and with no impact on the SMRs, missing data were excluded from the analysis. A sensitivity analysis was carried out by confining the study to the first admission for each patient during the study period.

Statistical significance was measured at the conventional 5% level. However, as this is affected by the differing sizes of the two study populations and the numbers of suicides, we arbitrarily defined suicide mortality following physical illnesses as ‘high mortality’ or ‘high risk’ for a statistically significant increased SMR >3, and as ‘increased mortality’ or ‘increased risk’ for a significantly increased SMR of between one and 3.

For physical illnesses with high suicide mortality, we investigated the extent to which the suicides risks were mediated by ‘pre-existing mental illness’. This was defined using mental and behavioural disorders recorded in any position on patients’ current inpatient records or from any previous acute admissions during the 9 year study period (see online Supplementary Appendix for these disorders and ICD-10 codes).

Logistic regression modelling was used to assess changes in suicide odds ratios (ORs) for each physical illness with high suicide mortality, firstly, after adjusting for pre-existing mental illness, patient age and sex alone (models 2) compared with age and sex alone (models 1). Secondly, after adjusting for pre-existing mental illness, year of discharge, social deprivation, age and sex (models 3) compared with age and sex (models 1). Social deprivation was measured in quintiles, based on the English Indices of Deprivation (Office for National Statistics, 2007) and corresponding Welsh Index of Multiple Deprivation (Welsh Assembly Government, 2008). The χ^2^ test was used to compare major categories of physical illnesses (circulatory, respiratory, gastrointestinal and all other) that were associated with suicide and emergency admissions respectively.

## Results

During the study period there were 11 004 389 acute admissions for physical illnesses across England and 713 496 across Wales, as defined in the methods section. These involved 8 970 811 and 577 474 different people respectively. The median age of the patients was 61.0 in England and 62.0 in Wales; 55.0% and 56.0%, respectively were female.

Within 1 year of discharge from hospital, there were a total of 1781 suicides in England (SMR = 1.7; 95% confidence interval [CI] 1.6–1.8) and 131 in Wales (SMR = 2.0; 1.7–2.3). The median age of the people who died from suicide was 56.0 in England and 53.0 in Wales and the percentages who were female were 30.2% and 28.2%, respectively.

### Suicide according to major physical illnesses

For each of the 48 physical illnesses included in the study, Table 1 shows the numbers of admissions and summary patient demographic details in both countries. The 48 physical illnesses were largely gastrointestinal disorders (17), circulatory (eight) or respiratory diseases (six), which were associated with respectively 23.8%, 18.6% and 15.3% of the study suicides. For comparison, all gastrointestinal, circulatory and respiratory diseases accounted for 17.4%, 20.4% and 13.9% of all acute admissions for physical illnesses (*p* < 0.001 for this comparison).

**Table 1.** Numbers of admissions and summary patient demographics for people hospitalised as emergencies for physical illnesses across England and Wales

	England			Wales		
Physical illness^a^	No. of admissions	Mean (and median) age at admission	% female	No. of admissions	Mean (and median) age at admission	% female
Infectious & parasitic diseases	284 174	50.0 (49.0)	52.2	17 274	54.3 (53.0)	55.0
Septicaemia	42 747	67.2 (71.0)	51.7	3260	67.6 (70.0)	54.8
Endocrine nutritional & metabolic diseases	279 099	60.0 (64.0)	51.6	18 347	60.7 (65.0)	52.2
Diabetes mellitus	121 531	52.5 (53.0)	41.8	8509	54.3 (56.0)	43.3
Hypoglycaemia	32 486	66.6 (72.0)	49.0	2088	66.6 (72.0)	50.1
Hypo-osmolality & hyponatraemia	23 897	75.1 (78.0)	71.5	1371	75.3 (79.0)	72.8
Cancers	459 021	68.0 (70.0)	48.3	31 267	69.4 (71.0)	47.3
Gastrointestinal cancers	118 641	70.1 (72.0)	43.7	8640	71.5 (73.0)	43.9
Lung cancer	65 334	70.3 (71.0)	43.7	4833	70.6 (71.0)	43.0
Lymphomas	58 551	63.8 (66.0)	43.9	3311	65.3 (68.0)	43.7
Disease of the nervous system	443 852	57.7 (59.0)	53.6	29 206	56.9 (62.0)	53.4
Parkinson's disease	15 997	76.5 (78.0)	38.9	1321	77.0 (78.0)	43.4
Epilepsy	104 316	47.8 (45.0)	44.3	5845	48.8 (47.0)	46.0
Transient ischaemic attack	117 949	72.0 (74.0)	50.1	6756	71.0 (74.0)	48.0
Circulatory diseases	2 247 914	68.4 (71.0)	44.5	145 660	69.1 (71.0)	45.4
Ischaemic heart disease	765 742	67.5 (68.0)	37.1	49 508	68.3 (69.0)	38.5
Stroke	337 752	73.1 (76.0)	48.7	21 041	73.4 (76.0)	48.6
Pulmonary embolism	87 602	63.5 (66.0)	52.6	5079	63.0 (66.0)	52.5
Atrial fibrillation	274 179	69.7 (72.0)	49.3	19 050	70.2 (72.0)	50.2
Heart failure	170 924	77.6 (79.0)	49.1	12 223	77.4 (79.0)	49.2
Phlebitis & thrombophlebitis	115 605	60.6 (63.0)	50.2	4879	62.1 (64.0)	51.5
Haemorrhoids	35 192	49.7 (46.0)	40.8	2796	50.5 (48.0)	40.6
Hypotension	57 460	77.4 (80.0)	54.5	3794	76.7 (79.0)	54.2
Respiratory diseases	1 530 397	61.2 (66.0)	52.3	105 950	62.4 (67.0)	53.3
Pneumonia	396 821	66.7 (71.0)	49.1	25 488	66.1 (70.0)	49.4
Acute lower respiratory infections	289 857	66.4 (72.0)	55.1	22 236	66.8 (72.0)	55.6
COPD	326 051	71.1 (72.0)	51.2	25 273	71.1 (72.0)	52.0
Asthma	177 629	47.4 (45.0)	65.9	10 911	49.5 (48.0)	68.8
Acute tonsillitis	57 472	29.5 (27.0)	53.5	3862	29.3 (26.0)	55.4
Pleural effusion	46 274	69.0 (72.0)	44.8	2698	70.0 (72.0)	44.2
Gastrointestinal diseases	1 918 700	55.6 (56.0)	52.0	130 016	56.6 (58.0)	53.2
Gastro-oesophageal reflux disease	46 786	59.3 (60.0)	50.2	3808	58.9 (60.0)	50.5
Peptic ulcer	75 321	63.7 (67.0)	40.1	4305	64.3 (68.0)	41.2
Gastritis	71 561	52.6 (51.0)	50.3	5872	53.2 (53.0)	50.7
Acute appendicitis	156 380	37.5 (34.0)	44.0	8733	37.3 (34.0)	41.9
Herniae	85 714	61.6 (64.0)	40.9	6119	61.9 (64.0)	40.3
Non-infective gastroenteritis	209 855	56.2 (58.0)	60.3	13 943	57.4 (60.0)	61.0
Intestinal obstruction	69 249	65.2 (68.0)	53.6	3969	65.7 (68.0)	54.1
Diverticular disease	99 882	66.7 (68.0)	61.4	7782	66.9 (68.0))	62.6
Alcoholic liver disease	31 347	51.4 (51.0)	35.0	2535	52.1 (52.0)	34.8
Other liver disease	22 200	57.2 (59.0)	48.7	1479	59.4 (61.0)	48.6
Gallstone disease	233 627	57.0 (58.0)	67.5	19 326	57.2 (59.0)	67.9
Acute pancreatitis	68 649	55.5 (56.0)	47.5	4197	56.9 (58.0)	46.8
Chronic pancreatitis	11 716	47.6 (46.0)	28.6	833	48.5 (48.0)	27.4
Upper gastrointestinal bleeding	134 706	59.3 (62.0)	42.0	7988	60.0 (63.0)	40.8
Constipation	115 470	63.1 (68.0)	60.1	9139	61.9 (68.0)	60.9
Chronic anal fissure	67 090	41.4 (40.0)	29.2	4085	42.6 (41.0)	30.6
Anal haemorrhage	63 386	63.3 (68.0)	52.0	1703	63.8 (68.0)	51.8
Skin diseases	558 074	51.9 (50.0)	45.6	33 158	52.9 (52.0)	46.1
Cellulitis	252 877	59.5 (60.0)	45.8	15 405	59.6 (61.0)	45.0
Cutaneous abscess, furuncle & carbuncle	129 863	42.0 (39.0)	45.2	6536	43.0 (40.0)	46.9
Musculoskeletal diseases	877 248	59.0 (60.0)	57.3	47 243	59.4 (61.0]	56.8
Back pain	179 564	52.1 (49.0)	56.6	10 053	53.2 (51.0)	56.8
Genitourinary diseases	1 172 753	53.9 (51.0)	59.9	74 484	53.8 (52.0)	61.7
Acute renal failure	58 879	72.4 (76.0)	44.5	3255	72.6 (76.0)	45.2
Urinary tract infections	399 831	68.4 (77.0)	68.3	26 004	65.8 (74.0)	69.8
Orchitis & epididymitis	38 399	42.3 (39.0)	0	3143	41.5 (38.0)	0
Diseases of the blood	180 963	64.2 (69.0)	62.0	11 693	67.5 (71.0)	62.8
Anaemias	134 796	66.9 (73.0)	62.7	8796	70.7 (75.0)	63.0
All physical illnesses	11 004 389	57.9 (61.0)	55.0	713 496	58.4 (62.0)	56.0

a Includes illnesses which led to at least eight suicides within 1 year of discharge from hospital.

Table 2 shows the numbers of suicides, corresponding SMRs and summary demographics for the people who died from suicide. Three diseases had high suicide mortality (significant SMR >3) in both England and Wales and 11 had high suicide mortality in one but not both countries (Table 2). These were gastrointestinal disorders (six), endocrine, nutritional & metabolic diseases (three), diseases of the nervous system (two) and respiratory, genitourinary and musculoskeletal diseases (one each). None of the 48 diseases had significantly decreased SMRs.

**Table 2.** Numbers of admissions and suicides with corresponding SMRs and summary patient demographics for people who died from suicide after discharge from hospital with physical illnesses across England and Wales

	England					Wales				
Physical illness^a^	No. of admissions	No. of suicides at 1 year^b^	SMR (95% CI)	Mean (and median) age at suicide	% female	No. of admissions	No. of suicides at 1 year^b^	SMR (95% CI)	Mean (and median) age at suicide	% female
Infectious & parasitic diseases	284 174	36	1.3 (0.9–1.7)	48.1 (41.0)	28	17 274	^c^	1.2 (0.1–3.5)	^c^	^c^
Septicaemia	42 747	8	1.9 (0.8–3.5)	60.8 (65.5)	25	3260	^c^	3.3 (0.0–13.0)	^c^	^c^
Endocrine, nutritional & metabolic diseases	279 099	71	2.6 (2.0–3.2)	52.9 (52.0)	35	18 347	7	3.9 (1.6–7.4)	46.1 (47.0)	0
Diabetes mellitus	121 531	31	2.3 (1.6–3.2)	47.8 (45.0)	32	8509	5	5.5 (1.7–11.4)	46.6 (47.0)	0
Hypoglycaemia	32 486	12	3.7 (1.9–6.2)	56.9 (55.5)	42	2088	^c^	4.9 (0.0–19.2)	^c^	^c^
Hypo-osmolality & hyponatraemia	23 897	10	5.6 (2.5–9.9)	66.9 (68.5)	30	1371	0	0	^c^	^c^
Cancers	459 021	87	2.0 (1.6–2.4)	67.4 (68.0)	17	31 267	6	2.0 (0.7–3.9)	75.5 (75.0)	0
Gastrointestinal cancers	118 641	24	2.0 (1.3–3.0)	66.5 (68.0)	4	8640	^c^	2.4 (0.2–6.8)	^c^	^c^
Lung cancer	65 334	15	2.4 (1.3–3.7)	69.5 (68.0)	20	4833	^c^	2.2 (0.0–8.5	^c^	^c^
Lymphomas	58 551	15	2.5 (1.4–3.9)	67.6 (69.0)	13	3311	^c^	0	^c^	^c^
Disease of the nervous system	443 852	98	2.2 (1.8–2.7)	54.2 (54.0)	38	29 206	9	3.2 (1.4–5.6)	51.9 (47.0)	33
Parkinson's disease	15 997	9	5.6 (2.5–9.9)	70.3 (71.0)	22	1321	0	0	^c^	^c^
Epilepsy	104 316	30	3.0 (2.0–4.1)	42.2 (41.5)	43	5845	^c^	4.7 (0.9–11.3)	^c^	^c^
Transient ischaemic attack	117 949	15	1.5 (0.9–2.4)	65.2 (67.0)	27	6756	^c^	3.1 (0.3–8.9)	^c^	^c^
Circulatory diseases	2 247 914	335	1.4 (1.3–1.6)	64.2 (65.0)	25	145 660	20	1.4 (0.8–2.0)	60.9 (63.5)	30
Ischaemic heart disease	765 742	107	1.3 (1.0–1.5)	64.1 (63.0)	17	49 508	^c^	0.4 (0.0–1.1)	^c^	^c^
Stroke	337 752	50	1.5 (1.0–2.0)	68.9 (70.0)	22	21 041	5	2.5 (0.8–5.2)	73.4 (71.0)	0
Pulmonary embolism	87 602	15	1.8 (1.0–2.8)	51.3 (55.0)	33	5079	0	0	^c^	^c^
Atrial fibrillation	274 179	35	1.3 (0.9–1.8)	72.7 (74.0)	31	19 050	^c^	1.6 (0.3–4.0)	^c^	^c^
Heart failure	170 924	18	1.1 (0.7–1.7)	76.9 (79.0)	33	12 223	^c^	0.8 (0.0–3.4)	^c^	^c^
Phlebitis & thrombophlebitis	115 605	19	1.6 (1.0–2.4)	50.2 (50.0)	16	4879	^c^	2.0 (0.0–8.0)	^c^	^c^
Haemorrhoids	35 192	9	2.1 (0.9–3.7)	54.0 (53.0)	22	2796	0	0	^c^	^c^
Hypotension	57 460	15	2.9 (1.6–4.5)	71.0 (77.0)	47	3794	^c^	2.9 (0.0–11.5)	^c^	^c^
Respiratory diseases	1 530 397	273	2.0 (1.7–2.2)	61.5 (63.0)	33	105 950	20	2.0 (1.3–3.0)	56.0 (56.5)	20
Pneumonia	396 821	54	1.4 (1.1–1.8)	61.8 (61.0)	28	25 488	8	3.1 (1.3–5.7)	63.9 (67.0)	38
Acute lower respiratory infections	289 857	42	1.5 (1.1–2.0)	65.9 (71.5)	31	22 236	^c^	1.0 (0.1–2.7)	^c^	^c^
COPD	326 051	79	2.8 (2.2–3.5)	68.0 (68.0)	42	25 273	^c^	1.8 (0.5–3.9)	^c^	^c^
Asthma	177 629	21	1.3 (0.8–2.0)	48.8 (48.0)	43	10 911	0	2.2 (0.2–6.2)	^c^	^c^
Acute tonsillitis	57 472	11	2.1 (1.0–3.4)	34.1 (35.0)	18	3862	^c^	2.9 (0.0–11.4)	^c^	^c^
Pleural effusion	46 274	13	2.8 (1.5–4.5)	72.5 (69.0)	8	2698	0	0	^c^	^c^
Gastrointestinal diseases	1 918 700	425	2.2 (2.0–2.4)	53.6 (52.0)	28	130 016	31	2.4 (1.6–3.3)	48.7 (42.0)	29
Gastro-oesophageal reflux disease	46 786	11	2.2 (1.1–3.7)	53.2 (57.0)	46	3808	0	0	^c^	^c^
Peptic ulcer	75 321	13	1.4 (0.7–2.4)	56.8 (56.0)	8	4305	0	0	^c^	^c^
Gastritis	71 561	33	4.4 (3.0–6.0)	50.0 (49.0)	24	5872	^c^	4.9 (1.0–12.4)	^c^	^c^
Acute appendicitis	156 380	17	1.0 (0.6–1.5)	37.1 (37.0)	18	8733	^c^	2.1 (0.2–6.0)	^c^	^c^
Herniae	85 714	17	1.8 (1.0–2.7)	60.8 (62.0)	6	6119	^c^	2.9 (0.3–8.4)	^c^	^c^
Non-infective gastroenteritis	209 855	42	2.2 (1.6–2.9)	55.9 (51.5)	48	13 943	^c^	0.8 (0.0–3.1)	^c^	^c^
Intestinal obstruction	69 249	11	1.7 (0.8–2.8)	66.8 (65.0)	36	3969	0	0	^c^	^c^
Diverticular disease	99 882	20	2.2 (1.3–3.3)	62.0 (63.5)	50	7782	^c^	2.9 (0.3–8.3)	^c^	^c^
Alcoholic liver disease	31 347	20	4.8 (2.9–7.1)	46.4 (47.5)	10	2535	^c^	6.0 (0.6–17.2)	^c^	^c^
Other liver disease	22 200	13	5.6 (3.0–9.0)	43.0 (45.0)	23	1479	^c^	6.6 (0.0–25.8)	^c^	^c^
Gallstone disease	233 627	16	0.8 (0.5–1.3)	56.1 (60.0)	63	19 326	0	0	^c^	^c^
Acute pancreatitis	68 649	18	2.4 (1.4–3.6)	45.3 (39.5)	33	4197	^c^	4.3 (0.4–12.5)	^c^	^c^
Chronic pancreatitis	11 716	13	7.7 (4.1–12.4)	43.5 (44.0)	8	833	^c^	8.3 (0.0–32.4)	^c^	^c^
Upper gastrointestinal bleeding	134 706	52	3.4 (2.5–4.4)	49.3 (48.0)	16	7988	^c^	4.5 (1.2–10.0)	^c^	^c^
Constipation	115 470	43	4.1 (2.9–5.4)	66.1 (68.0)	33	9139	6	7.5 (2.7–14.2)	62.2 (65.5)	67
Chronic anal fissure	67 090	10	1.1 (0.5–1.8)	36.4 (37.0)	10	4085	^c^	1.8 (0.0–7.0)	^c^	^c^
Anal haemorrhage	63 386	13	2.1 (1.1–3.4)	61.7 (59.0)	23	1703	0	0	^c^	^c^
Skin diseases	558 074	96	1.5 (1.3–1.9)	48.7 (44.0)	23	33 158	9	2.5 (1.1–4.4)	57.0 (51.0)	22
Cellulitis	252 877	46	1.6 (1.2–2.2)	49.0 (43.5)	17	15 405	^c^	2.3 (0.6–5.2)	^c^	^c^
Cutaneous abscess, furuncle & carbuncle	129 863	29	1.9 (1.3–2.7)	42.8 (41.0)	21	6536	^c^	4.1 (0.8–10.0)	^c^	^c^
Musculoskeletal diseases	877 248	146	1.7 (1.5–2.0)	56.4 (54.0)	34	47 243	9	2.0 (0.9–3.4)	53.0 (54.0)	33
Back pain	179 564	46	2.5 (1.8–3.3)	42.8 (49.0)	48	10 053	^c^	3.9 (1.0–8.8)	^c^	^c^
Genitourinary diseases	1 172 753	130	1.2 (1.0–1.4)	50.8 (45.0)	39	74 484	15	2.3 (1.3–3.6)	56.0 (57.0)	47
Acute renal failure	58 879	10	1.7 (0.8–2.9)	59.4 (58.5)	30	3255	^c^	3.1 (0.0–12.3)	^c^	^c^
Urinary tract infections	399 831	41	1.3 (0.9–1.7)	58.2 (60.0)	54	26 004	7	3.5 (1.4–6.5)	60.9 (64.0)	71
Orchitis & epididymitis	38 399	8	1.3 (0.5–2.3)	44.9 (37.0)	0	3143	^c^	2.0 (0.0–7.7)	^c^	0
Diseases of the blood	180 963	29	1.8 (1.2–2.6)	58.7 (54.0)	24	11 693	^c^	2.0 (0.2–5.8)	^c^	^c^
Anaemias	134 796	21	1.7 (1.1–2.6)	62.2 (57.0)	33	8796	^c^	2.3 (0.3–7.8)	^c^	^c^
All physical illnesses	11 004 389	1781	1.7 (1.6–1.8)	57.0 (56.0)	30	713 496	131	2.0 (1.7–2.3)	55.1 (53.0)	28

a Includes illnesses which led to at least eight suicides within 1 year of discharge from hospital.

b Includes suicides or injury & poisoning of undetermined intent, recorded as the underlying cause of death.

c Denotes less than five suicides.

The three diseases with high suicide mortality in both countries were gastrointestinal disorders: constipation (SMR = 4.1 in England and 7.5 in Wales); gastritis (SMRs = 4.4 and 4.9 respectively); and upper gastrointestinal bleeding (3.4 and 4.5). The suicide SMR of 3.5 for gastritis in England increased to 11.7 for cases confined to alcoholic gastritis. The three gastrointestinal diseases with high suicide mortality in one country were alcoholic liver disease (SMR = 4.8, England), other liver disease (5.6, England) and chronic pancreatitis (7.7, England). For each of these three disorders the corresponding SMRs were >3 in Wales although, with few suicides involved, none were significant.

Three endocrine, nutritional & metabolic diseases with high suicide risks in one country were diabetes (SMR = 5.5, Wales), hypoglycaemia (SMR = 3.7, England) and hypo-osmolality & hyponatraemia (SMR = 5.6, England).

Two diseases of the nervous system with high suicide mortality in one country were epilepsy (SMR = 3.0, England) and Parkinson's disease (SMR = 5.6, England). The SMR for epilepsy in Wales was also high (4.7) but marginally not significant. The remaining disorders with high suicide mortality in either country were pneumonia (SMR = 3.1, Wales), urinary tract infections (SMR = 3.5, Wales) and back pain (SMR = 3.9, Wales).

When assessing suicide risks according to major disease grouping (ICD chapter) the highest SMRs were for endocrine, nutritional & metabolic diseases (SMR = 2.6, England and 3.9, Wales), diseases of the nervous system (2.2, England and 3.2, Wales) and gastrointestinal disorders (2.2, England and 2.4, Wales). More moderately increased suicide rates were apparent for cancers, circulatory, respiratory, genitourinary, skin, blood and musculoskeletal diseases, with no increased risk for infectious diseases.

### Suicide according to patient demographics

Median ages at suicide were lowest following acute tonsillitis (35), orchitis and epididymitis (37), cutaneous abscess, furuncle & carbuncle (41), epilepsy (41.5), cellulitis (43.5), diabetes (45) and various gastrointestinal disorders, including acute appendicitis (37), chronic anal fissure (37), acute pancreatitis (39.5), chronic pancreatitis (44), other liver disease (45), and alcoholic liver disease (47.5; Table 2). Median ages at suicide were highest (⩾70 years) for Parkinson's disease and various circulatory or respiratory disorders including heart failure, stroke, atrial fibrillation, hypotension and acute lower respiratory infections.

### Suicides following admissions for constipation

There were 49 suicides within 1 year of discharge with constipation (mean patient age = 65.6 years; s.d. = 18.5). The majority (27; 55%) were aged 65+ years, 15 (31%) were 45–64 years, six (12%) were 25–44 years and one was <25 years. The underlying causes of death were hanging, strangulation or suffocation (20), self poisoning (12), drowning (6) and other forms of intentional or undetermined self harm (11). The most frequently recorded secondary or subsidiary causes of death (>5 cases) were asphyxia (20), drug poisoning (15), depression (9), multiple injuries (6) and cancers (5). The most frequently recorded co-morbidities on the index inpatient records were hypertension (9), depression (7), ischaemic heart disease (6), intestinal diseases (6) followed by (<5 cases) asthma, personal history of psychoactive substance abuse, personal history of genitourinary or circulatory diseases, nausea and vomiting, retention of urine and hypercholesterolaemia.

### Sensitivity analysis

The sensitivity analysis, confined to first study admissions for each patient, is summarised in an online Supplementary Table for all physical illnesses with high significant SMRs >3. This shows little change in the study findings with no significant differences in SMRs.

### Influence of factors including pre-existing mental illness

For most physical illnesses associated with high suicide risks, additional adjustment for pre-existing mental illness had little or moderate influence on suicide ORs (<35% reduction; Table 3). However, there were larger reductions for alcoholic liver disease (55% reduction) and chronic pancreatitis (43%) in England. The prevalence of pre-existing mental illness was higher for alcoholic liver disease (67%) and chronic pancreatitis (43%) than for other physical illnesses (<30%; Table 3). There was little additional confounding influence for social deprivation and year.

**Table 3.** Adjustment of suicide odds ratios for (a) pre-existing mental illness, and (b) pre-existing mental illness, calendar year and social deprivation (as well as patient age and sex): for physical illnesses associated with high suicide risks in England or Wales

Physical illness	% of cases with recorded pre-existing mental illness	Models 1 (adjusted for patient age and sex) Odds ratio (95% CI)	Models 2 (adjusted for pre-existing mental illness, age and sex) Odds ratio (95% CI)	% reduction in suicide odds ratio (models 2 *v.* 1)	Models 3 (adjusted for pre-existing mental illness, year, social deprivation, age and sex) Odds ratio (95% CI)	% reduction in suicide odds ratio (models 3 *v.* 1)
England						
Hypoglycaemia	11.5	2.27 (1.28–4.00)	2.07 (1.17–3.65)	8.8	2.11 (1.20–3.73)	7.0
Hypo-osmolality & hyponatraemia	18.6	3.42 (1.83–6.37)	2.50 (1.34–4.66)	26.9	2.52 (1.32–4.70)	26.3
Parkinson's disease	12.4	3.53 (1.83–6.82)	2.82 (1.40–5.66)	20.1	2.41 (1.15–5.07)	31.7
Epilepsy	27.5	1.78 (1.24–2.56)	1.23 (0.86–1.77)	30.9	1.15 (0.79–1.67)	35.4
Gastritis	17.1	2.63 (1.86–3.71)	2.07 (1.46–2.94)	21.3	2.04 (1.43–2.91)	22.4
Alcoholic liver disease	66.6	2.67 (1.72–4.16)	1.19 (0.76–1.87)	55.4	1.16 (0.74–1.81)	56.6
Other liver disease	15.3	3.20 (1.86–5.53)	2.55 (1.45–4.51)	20.3	2.38 (1.31–4.31)	25.6
Chronic pancreatitis	43.3	4.56 (2.64–7.89)	2.60 (1.50–4.51)	43.0	2.59 (1.50–4.49)	43.2
Upper gastrointestinal bleeding	18.8	2.11 (1.60–2.79)	1.60 (1.21–2.12)	24.2	1.56 (1.17–2.07)	26.1
Constipation	9.2	2.48 (1.83–3.36)	2.43 (1.79–3.29)	2.0	2.47 (1.83–3.35)	0.4
Wales						
Diabetes mellitus	10.3	2.68 (1.10–6.58)	2.39 (0.97–5.87)	10.8	2.44 (1.00–6.00)	9.0
Gastritis	13.9	2.39 (0.76–7.51)	1.81 (0.57–5.71)	24.2	1.87 (0.59–5.90)	21.8
Upper gastrointestinal bleeding	17.6	2.31 (0.85–6.26)	1.51 (0.55–4.13)	34.6	1.17 (0.37–3.71)	49.4
Constipation	8.2	4.15 (1.83–9.44)	4.07 (1.79–9.27)	1.9	4.11 (1.80–9.43)	0.9
Pneumonia	10.5	1.74 (0.85–3.58)	1.53 (0.75–3.14)	12.1	1.54 (0.75–3.16)	11.5
Back pain	5.4	1.93 (0.71–5.23)	2.25 (0.83–6.11)	−16.6	2.35 (0.85–6.26)	−21.8
Urinary tract infections	7.4	2.00 (0.93–4.32)	1.97 (0.91–4.26)	1.5	1.97 (0.91–4.28)	1.5

## Discussion

The study was designed to investigate all possible physical illnesses that may be associated with substantial suicide mortality, with very little or nothing previously reported on possible links with suicide for many of these illnesses. These include gastritis, chronic pancreatitis, upper gastrointestinal bleeding, acute tonsillitis, pleural effusion, hypoglycaemia and hypo-osmolality & hyponatraemia, so that many of the study findings are novel.

The study found little or no increased suicide mortality following acute admissions for most of the 48 physical illnesses investigated. However, it found that most evidence of high suicide mortality (significant SMR >3) was, firstly, for gastrointestinal diseases that are often alcohol-related (liver disease, chronic pancreatitis, gastritis and upper gastrointestinal bleeding) and other diseases that are sometimes alcohol-related (hypo-osmolality & hyponatraemia and pneumonia). Secondly, for conditions which are often caused by certain medications (constipation) or have suicide risks that are often linked to various therapeutic medications (epilepsy and Parkinson's disease). Thirdly, for certain endocrine disorders that relate acutely to poor blood glucose control (diabetes and hypoglycaemia).

There was a strong and consistent increased risk of suicide (four-fold in England and seven-fold in Wales) for patients discharged with constipation. Although this high suicide risk for constipation has not been quantified previously, severe constipation is common in people taking opioids or with opioid dependence and a known adverse effect for certain medications used for treating anaemia, epilepsy, pain, depression and other mental disorders. These medications include aluminium antacids (Talley *et al.* 2003), some antiepileptics (Jahromi *et al.* 2011), iron and calcium supplements (Talley *et al.* 2003; Jahromi *et al.* 2011; Tolkien *et al.* 2015), opiate analgesics (Talley *et al.* 2003; Benyamin *et al.* 2008; Camilleri, 2011; Rauck *et al.* 2017), some antidepressants (Talley *et al.* 2003; Watanabe *et al.* 2010) and antipsychotics, particularly clozapine (De Hert *et al.* 2011; Baptista *et al.* 2015; Shirazi *et al.* 2016). The link between these medications and disorders (some of which may be linked with alcohol misuse or heavy alcohol consumption) associated with high risks of suicide may account for this association.

We found evidence of an elevated suicide mortality of four- to eight-fold for several gastrointestinal disorders that are either wholly or partially linked with alcohol misuse or heavy alcohol consumption, particularly among younger people. These disorders were gastritis, alcoholic liver disease, chronic pancreatitis and upper gastrointestinal bleeding. The suicide risk for gastritis in England was increased further (11.7-fold) for cases of alcoholic aetiology. A previous cohort study reported 3.6-fold increased suicide mortality for liver disease overall (Erlangsen *et al.* 2015), although there have been no previous reports on suicide risks for gastritis, chronic pancreatitis or upper gastrointestinal bleeding. Links between suicide and both alcohol misuse disorder and heavy alcohol consumption are well established (Britton & McPherson, 2001; Wilcox *et al.* 2004; Schneider *et al.* 2011) with a recent meta analysis citing suicide SMRs of 9.8 and 3.5, respectively, for these two disorders (Britton & McPherson, 2001). Alcohol consumption or misuse accounts for about 70% of cases of chronic pancreatitis and high minorities of both gastritis and upper gastrointestinal bleeding cases, the latter in particular through bleeds caused by varices, Mallory-Weiss tear, gastritis and oesophageal cancer (Williams *et al.* 2007). The high SMR for other liver disease may also involve some cases that were alcohol related but not recorded. As well as a strong link between alcohol misuse and suicide, attempted suicides have also been documented occasionally in case reports of gastritis (Wu *et al.* 2001; Descatha *et al.* 2009), pancreatitis (Hantson & Mahieu, 2000) and upper gastrointestinal bleeding (Zhao *et al.* 2010; Altay *et al.* 2012), disorders that can each arise through complications of medicinal intoxication or poisoning during suicide attempts.

There was evidence of high suicide mortality for several major endocrine or metabolic diseases; diabetes, hypoglycaemia, and hypo-osmolality & hyponatraemia. Suicide mortality was increased 5.5-fold for diabetes in Wales and 2.3-fold in England. Previous disease register studies of type I diabetes from Scandinavia have cited suicide SMRs of 2.0 (Wibell *et al.* 2001), and 1.6 (Kyvik *et al.* 1994), while an English study of hospitalised diabetes among young people reported a much higher SMR of 11.7 (Roberts *et al.* 2004). Our more intermediate SMRs of 5.5 and 2.3 would be increased through being based on hospitalised cases, but would be decreased through including type 2 with type 1 diabetes. Suicide risks in diabetes are inter linked with depression, poor compliance with diabetes treatment and blood glucose control (Lustman *et al.* 2000; Sarkar & Balhara, 2014), which would explain some of the selection effects towards higher suicide mortality in studies based on inpatient cases. It would also partly explain our high suicide rate for hypoglycaemia (3.7 in England). We also found high suicide mortality (SMR = 5.6 in England) for hypo-osmolality & hyponatraemia, which has not been reported previously, can cause acute confusional states and may also be linked to cases of alcoholic aetiology.

We found high suicide mortality in England for two diseases of the nervous system; epilepsy and Parkinson's disease. There are well established links with suicide and co-morbid depression for both epilepsy (Hawton *et al.* 1980; Bell *et al.* 2009*a*, *b*; Singhal *et al.* 2014; Erlangsen *et al.* 2015) and Parkinson's disease (Erlangsen *et al.* 2015). For epilepsy, a recent meta analysis reported a suicide SMR of 3.3 (Bell *et al.* 2009*a*, *b*), which is consistent with our SMRs of 3.0 in England and 4.7 in Wales. For both Parkinson's disease and epilepsy, treatments including various drugs (Bell *et al.* 2009*a*, *b*; Andersohn *et al.* 2010), and deep brain simulation (Krack *et al.* 2003; Appleby *et al.* 2007), have been associated with increased risks of suicide, although the evidence is often quite weak and conflicting.

There was little or no evidence of substantially increased suicide mortality in the year following hospital discharge for most circulatory and respiratory diseases, with the exception of pneumonia in Wales (SMR = 3.1) although the SMR was 1.4 in England. There is little literature on an association between pneumonia and suicide, although excessive and prolonged use of alcohol is linked with increased hospital admissions for pneumonia (Kornum *et al.* 2012) while increased suicides have also been reported following hospitalisation with various infections (Lund-Sørensen *et al.* 2016). Suggested mechanisms for the links between infections and suicide include proinflammatory cytokines, inflammatory metabolites, and effects of antibiotic treatments on the biome (Lund-Sørensen *et al.* 2016). Some studies have found evidence of increased suicide risks for both cardiovascular and cerebrovascular diseases, but effect sizes are variable and often small or not significantly increased (Placido & Sposito, 2009; Larsen *et al.* 2010; Pompili *et al.* 2012; Webb *et al.* 2012; Crump *et al.* 2014; Singhal *et al.* 2014; Erlangsen *et al.* 2015). Another study reported low suicide risks in a cohort of inpatients with acute general physical disorders (Olfson *et al.* 2016). We also found high suicide mortality for urinary tract infections in Wales (SMR = 3.5) but not in England (1.3). There are reported links between suicide and interstitial cystitis and bladder pain syndrome (Ratner, 2001; Hepner *et al.* 2012).

We found modest increased SMRs for cancers (2.0 in both England and Wales), which are comparable or slightly higher than those from large disease register studies in England (1.7; Robinson *et al.* 2009), Sweden (1.5; Bjorkenstam *et al.* 2005), Norway (1.6; Hem *et al.* 2004), Denmark (1.7; Yousaf *et al.* 2005), Italy (1.5; Crocetti *et al.* 2012), and Austria (1.2; Vyssoki *et al.* 2015).

For most physical illnesses with high suicide mortality, there was moderate or little reduction in suicides (<35%) after additional adjustment for pre-existing mental illness and also low prevalence of mental illness (<30%). Although our case-ascertainment of pre-existing mental illness from secondary diagnoses in acute national inpatient data is incomplete, the findings indicate that subsequent suicides are largely independent of mental illness for physical illnesses with low mental illness prevalence. However, the two physical illnesses with high suicide risks that were most strongly mediated by pre-existing mental illness were alcoholic liver disease (55% reduction in risk) and chronic pancreatitis (43%). These two illnesses had by far the highest recorded prevalence of mental illness, largely mental and behavioural disorders through psychoactive substance use, mainly alcohol.

Major strengths are that this is one of the largest population studies of suicide after discharge from hospital, based on more than 1900 suicides among a total hospitalised population of almost 12 million people. It provides large-scale evidence on suicide risks for all major physical illnesses, which were associated with at least eight suicides, including many conditions that have not been reported previously. Importantly for confirmatory purposes, it is based on two independently collected information sources, although processes for death certification and inpatient data collection are similar in both countries. It is also based on validated record linkage methodology that has been used extensively in previous follow-up mortality studies, which systematically identifies all deaths following discharge from hospital through national death certificate data. Although the inpatient data sources are confined to public hospitals, these account for almost all acute admissions. When assessing increased suicide risks, ORs obtained through logistic regression modelling were typically lower (although not significantly so) than corresponding suicide SMRs, as they measure increased suicide risks with respect to different comparative reference points. SMRs are used more routinely to highlight increased risks of suicide (especially by government agencies) but do not readily facilitate adjustment for additional factors in this study, such as pre-existing mental illness.

Study limitations are that the national administrative inpatient data lacks detailed information about therapeutic treatment and prescribed medications as well as details of disease history and severity, while the principal diagnosis used to identify physical diseases is not accurate in all cases (Capital, 2014). Further, the diagnostic categories used for the different physical illnesses are constrained by the limitations of ICD coding. For example, when studying diabetes mellitus, we were not able to distinguish type 1 from type 2 diabetes in most cases. The definition of physical illness used here is based on acute admission so excludes people with more mild conditions that might appear in studies based on primary care data, disease registers, general population cohorts, outpatient or elective admissions. However, by restricting the study to acute admissions, it excludes a large volume of elective admissions that are often for diagnostic investigations rather than for active or present disease (Li & Rothwell, 2016). There was also much less study power in Wales than in England.

Despite these limitations, the sizes of the populations studied and the consistency of the findings across the two countries suggests that the study findings are valid and highlight potential opportunities for suicide prevention. Associations between physical illnesses and suicide can be mediated in some cases by mental illnesses, which we measured from diagnoses recorded in patients’ current or previous acute admissions. As mental illnesses are often not recorded comprehensively as secondary diagnoses in this inpatient data, further research should focus on more in-depth, smaller-scale studies that incorporate full mental health or primary care diagnostic data, to establish the complex relationships between physical illnesses, mental illnesses and the subsequent risks of suicide.

### Preventative measures and policy implications

A recent large record linkage study of general and psychiatric hospital discharge across the UK (Dougall *et al.* 2014), found that those who died by suicide were 3.1 times more frequently discharged from general rather than psychiatric hospitals. While this study highlighted that engaging in suicide prevention within the general hospital setting deserves more attention, other than for self harm, it provided little evidence for where these efforts should be focussed. Delivering effective universal suicide prevention in this setting is a challenge given the large numbers of individuals admitted, the small number who will take their own lives and the range of specialties from which they are discharged. A more appropriate universal intervention could be on raising awareness of mental health issues in this setting. Selective interventions aimed at those discharged following acute hospital admissions can be considered to be a more focussed opportunity for intervention than for all admissions. Absolute risks of suicide are very low for almost all physical illnesses, which have major cost-effectiveness implications for potential interventions. Nonetheless, efforts targeted carefully at those discharged with constipation, some alcohol-related gastrointestinal and metabolic disorders, epilepsy and Parkinson's disease may yield the most benefits.

The types of activities that might reduce the risk of suicide include: raising awareness of suicidal behaviours to healthcare staff through education and training; ensuring that questions relating to mental health are asked and any suicidal ideation is elicited; improving communication of these issues between clinicians at the interface between primary and secondary care, between different specialities and at any outpatient appointments; improvements in psychiatric liaison services in general hospital settings; appropriate signposting to organisations offering support for particular conditions and/or for suicidal ideation and behaviours; and finally promoting social support by family members, carers or friends if appropriate.

## Conclusions

In summary, we found that emergency hospitalisations for most physical diseases usually lead to small or no increased risks of suicide in the year following discharge from hospital. However, for certain conditions, there is evidence of substantially increased risks of suicide. This is the case for several alcohol-related gastrointestinal disorders, some metabolic disorders, epilepsy, Parkinson's disease, constipation and a few other disorders. The National Institute for Health and Care Excellence currently recommends psychosocial assessment for people admitted to general hospitals for self harm. Further evidence is needed to determine the potential of psychosocial assessment for people admitted with alcohol and other high-risk conditions. At the very least for those conditions with high associated risks of suicide, healthcare staff should ensure they ask those admitted as emergencies questions relating to their mental health and whether they have any thoughts of suicide.

## Supplementary material

For supplementary material accompanying this paper visit http://dx.doi.org/10.1017/S0033291717001787. click here to view supplementary material
