# Supplementary material for: Suicide following acute admissions for physical illnesses across England and Wales
Source: Psychol Med. 2017 Jul 17;48(4):578–91. doi: 10.1017/S0033291717001787 (PMC5964467; doi:10.1017/S0033291717001787)
Supplement: Supplementary file 1 [file S0033291717001787sup001.zip › S0033291717001787sup001.docx]

**Appendix ICD-10 codes for the 48 physical illnesses included in the study**

|  |  |
| --- | --- |
| Infectious & parasitic diseases | A00-B99 |
| - Septicaemia | A40, A41 |
|  |  |
| Endocrine nutritional & metabolic diseases | E00-E90 |
| - Diabetes mellitus | E10-E14 |
| - Hypoglycaemia | E16.2 |
| - Hypo-osmolality & hyponatraemia | E87.1 |
|  |  |
| Cancers | C00-C97 |
| - Gastrointestinal cancers | C15-C26 |
| - Lung cancer | C33, C34 |
| - Lymphomas | C81-C96 |
|  |  |
| Disease of the nervous system | G00-G99 |
| - Parkinson's disease | G20 |
| - Epilepsy | G40, G41 |
| - Transient ischaemic attack | G45.9 |
|  |  |
| Circulatory diseases | I00-I99 |
| - Ischaemic heart disease | I20-I25 |
| - Stroke | I61-I64 |
| - Pulmonary embolism | I26 |
| - Atrial fibrillation | I48 |
| - Heart failure | I50 |
| - Phlebitis & thrombophlebitis | I80 |
| - Haemorrhoids | I84 |
| - Hypotension | I95 |
|  |  |
| Respiratory diseases | J00-J99 |
| - Pneumonia | J12-J18 |
| - Acute lower respiratory infections | J20-J22 |
| - COPD | J40-J44 |
| - Asthma | J45, J46 |
| - Acute tonsillitis | J03 |
| - Pleural effusion | J90 |
|  |  |
| Gastrointestinal diseases | K00-K93 |
| - Gastro-oesophageal reflux disease | K21 |
| - Peptic ulcer | K25-K28 |
| - Gastritis | K29.0-K29.7 |
| - Acute appendicitis | K35 |
| - Herniae | K40-K43, K45, K46 |
| - Noninfective gastroenteritis | K52 |
| - Intestinal obstruction | K56 |
| - Diverticular disease | K57 |
| - Alcoholic liver disease | K70 |
| - Other liver disease | K71-K77 |
| - Gallstone disease | K80 |
| - Acute pancreatitis | K85 |
| - Chronic pancreatitis | K86.0, K86.1 |
| - Upper gastrointestinal bleeding | I85.0,K22.6,K22.8,K25.0,K25.2,K25.4,K25.6,K26.0,K26.2,K26.4,K26.6,K27.0 |
|  | K27.2,K27.4,K27.6,K28.0,K28.2,K28.4,K28.6,K29.0,K92.0,K92.1,K92.2* |
| - Constipation | K59 |
| - Chronic anal fissure | K61.0 |
| - Anal haemorrhage | K62.5 |
|  |  |
| Skin diseases | L00-L99 |
| - Cellulitis | L03 |
| - Cutaneous abscess, furuncle & carbuncle | L02 |
|  |  |
| Musculoskeletal diseases | M00-M99 |
| - Back pain | M54 |
|  |  |
| Genitourinary diseases | N00-N99 |
| - Acute renal failure | N17 |
| - Urinary tract infections | N39.0 |
| - Orchitis & epididymitis | N45 |
|  |  |
| Diseases of the blood | D50-D99 |
| - Anaemias | D50-D64 |
|  |  |
| All physical diseases | A00-E99, G00-Q99 |
|  |  |

* Unless present with a diagnosis of lower gastrointestinal disease (C17.1-C21, K50-K52, K55-K57 or K60-K63)

**Appendix cont’d Mental and behavioural disorders (with ICD-10 codes) used to measure pre-existing mental illness**

| Mental and behavioural disorders due to psychoactive substance use | F10-F19 |
| --- | --- |
| Schizophrenia, schizotypal and delusional disorders | F20-F29 |
| Mood (affective) disorders | F30-F39 |
| Neurotic, stress-related and somatoform disorders | F40-F49 |
| Disorders of adult personality and behaviour | F60-F69 |
| Mental retardation | F70-F79 |
| Disorders of psychological development | F80-F89 |
| Behavioural and emotional disorders with onset that usually occurs in childhood and adolescence | F90-F99 |
