# Supplementary material for: Suicide following acute admissions for physical illnesses across England and Wales
Source: Psychol Med. 2017 Jul 17;48(4):578–91. doi: 10.1017/S0033291717001787 (PMC5964467; doi:10.1017/S0033291717001787)
Supplement: Supplementary file 1 [file S0033291717001787sup001.zip › S0033291717001787sup002.docx]

**Supplementary Table Sensitivity analysis based on first admissions only: numbers of admissions and suicides with corresponding SMRs and summary patient demographics for people who died from suicide after discharge from hospital with physical illnesses across England and Wales**

|  | **England** |  | **Wales** |
| --- | --- | --- | --- |

| **Physical illness ‡** | **No. of admissions** | **No. of suicides at one year †** | **SMR** | **(95% CI)** | **Mean**  **(and median) age at suicide** | **% female** |  | **No. of admissions** | **No. of suicides at one year †** | **SMR** | **(95% CI)** | **Mean**  **(and median) age at suicide** | **% female** |
| --- | --- | --- | --- | --- | --- | --- | --- | --- | --- | --- | --- | --- | --- |
|  |  |  |  |  |  |  |  |  |  |  |  |  |  |
| Endocrine, nutritional & metabolic diseases |  |  |  |  |  |  |  |  |  |  |  |  |  |
| - Diabetes mellitus | 92 200 | 20 | **1.9** | (1.2-2.9) | 47.8 (45.0) | 32% |  | 6392 | * | **4.3** | (0.8-10.7) | 46.6 (47.0) | 0% |
| - Hypoglycaemia | 22 804 | 10 | **4.4** | (2.1-7.6) | 56.9 (55.5) | 42% |  | 1450 | * | **7.0** | (0.0-27.6) | * | * |
| - Hypo-osmolality & hyponatraemia | 18 926 | 10 | **7.0** | (3.4-12.1) | 66.9 (68.5) | 30% |  | 1084 | 0 | **0** |  | * | * |
|  |  |  |  |  |  |  |  |  |  |  |  |  |  |
| Disease of the nervous system |  |  |  |  |  |  |  |  |  |  |  |  |  |
| - Parkinson's disease | 12 320 | 9 | **7.3** | (3.3-12.9) | 70.3 (71.0) | 22% |  | 1001 | 0 | **0** |  | * | * |
| - Epilepsy | 67 262 | 25 | **3.3** | (2.1-4.7) | 42.2 (41.5) | 43% |  | 4425 | * | **6.1** | (1.2-15.0) | * | * |
|  |  |  |  |  |  |  |  |  |  |  |  |  |  |
| Gastrointestinal diseases |  |  |  |  |  |  |  |  |  |  |  |  |  |
| - Gastritis | 66 634 | 25 | **4.3** | (2.9-6.0) | 50.0 (50.0) | 24% |  | 4941 | * | **5.8** | (1.1-14.3) | * | * |
| - Alcoholic liver disease | 24 714 | 19 | **5.7** | (3.4-8.6) | 46.4 (47.5) | 10% |  | 1988 | * | **7.6** | (0.7-21.9) | * | * |
| - Other liver disease | 18 410 | 11 | **5.7** | (2.8-9.5) | 43.0 (45.0) | 23% |  | 1227 | * | **7.9** | (0.0-25.8) | * | * |
| - Chronic pancreatitis | 8151 | 10 | **8.5** | (4.0-14.5) | 43.5 (44.0) | 8% |  | 554 | * | **12.3** | (0.0-48.2) | * | * |
| - Upper gastrointestinal bleeding | 111 551 | 44 | **3.4** | (2.5-4.6) | 49.3 (48.0) | 16% |  | 6548 | * | **5.4** | (1.4-12.1) | * | * |
| - Constipation | 92 890 | 31 | **3.7** | (2.5-5.2) | 66.1 (68.0) | 33% |  | 7100 | 5 | **8.0** | (2.5-16.6) | 62.2 (65.5) | 67% |
|  |  |  |  |  |  |  |  |  |  |  |  |  |  |
| Other diseases |  |  |  |  |  |  |  |  |  |  |  |  |  |
| - Pneumonia | 298 853 | 46 | **1.5** | (1.1-2.0) | 61.8 (61.0) | 28% |  | 19 349 | 6 | **3.1** | (1.1-6.0) | 63.9 (67.0) | 38% |
| - Back pain | 149 891 | 35 | **2.3** | (1.6-3.1) | 42.8 (49.0) | 48% |  | 8243 | * | **3.5** | (0.7-8.7) | * | * |
| - Urinary tract infections | 301 406 | 31 | **1.3** | (0.9-1.8) | 58.2 (60.0) | 54% |  | 19 893 | * | **2.0** | (0.4-4.8) | 60.9 (64.0) | 71% |
|  |  |  |  |  |  |  |  |  |  |  |  |  |  |
| All physical illnesses | 8 970 811 | 1450 | **1.7** | (1.6-1.8) | 56.4 (56.0) | 29% |  | 577 474 | 102 | **1.9** | (1.5-2.3) | 54.7 (53.0) | 25% |
|  |  |  |  |  |  |  |  |  |  |  |  |  |  |

**Notes**

This Table includes physical illnesses associated with high suicide risks in either England or Wales

* Denotes less than five suicides

‡ Includes illnesses which led to at least eight suicides within one year of discharge from hospital

† Includes suicides or injury & poisoning of undetermined intent, recorded as the underlying cause of death
